# Kidney and Metabolic Phenotypes in Glycogen Storage Disease Type-I Patients

**DOI:** 10.3389/fped.2020.00591

**Published:** 2020-09-11

**Authors:** Bilal Aoun, Sami Sanjad, Jad A. Degheili, Abir Barhoumi, Amina Bassyouni, Pascale E. Karam

**Affiliations:** ^1^Division of Pediatric Nephrology, Department of Pediatrics and Adolescent Medicine, American University of Beirut Medical Center, Beirut, Lebanon; ^2^Division of Urology, Department of Surgery, American University of Beirut Medical Center, Beirut, Lebanon; ^3^Department of Nutrition, American University of Beirut Medical Center, Beirut, Lebanon; ^4^Inherited Metabolic Diseases Program, Department of Pediatrics and Adolescent Medicine, American University of Beirut Medical Center, Beirut, Lebanon

**Keywords:** glycogen storage disease type I, microalbuminuria, angiotensin, hyperalaninemia, hypercalciuria, hypocitraturia, kidney disease

## Abstract

**Patients and Methods:** A retrospective chart review of 32 GSD- I patients, followed at the American University of Beirut Medical Center, between 2007 and 2018 was conducted. Diagnosis was confirmed by enzymatic and/or genetic studies. Clinical presentation, growth, and kidney outcome were assessed. All patients were evaluated for body mass index, blood parameters of metabolic control including uric acid, alanine, lactic acid, and triglycerides in blood. Kidney evaluation included creatinine clearance, microalbuminuria, citraturia, and calciuria as well as urine microalbumin/creatinine ratio.

**Results:** Almost one third of GSD-I patients developed microalbuminuria. This was detected below 7 months of age in 36% of patients who required early treatment with ACEI with significant reduction in albuminuria. Kidney stones were present in 6% and were associated with hypercalciuria and hypocitraturia. Poor metabolic control reflected by hyperuricemia, lactic acidosis, and hyperalaninemia were noted only in patients who developed microalbuminuria.

**Conclusion:** Glomerular injury may appear in early infancy in poorly controlled patients. Adequate metabolic control and ACEI therapy may improve kidney outcome in GSD I patients. Plasma alanine appears to be a promising and reliable marker reflecting metabolic control in GSD-I patients.

Glycogen storage disease type I (GSD- I) is a rare autosomal recessive disorder of glycogen and fat metabolism, leading to progressive accumulation of glycogen in major organs such as liver and kidneys. Variable stages of kidney disease from proximal and distal tubulopathies to irreversible glomerular injury may affect the outcome of GSD-I patients. Early detection of kidney complications, such as microalbuminuria, treatment with angiotensin converting enzyme inhibitors (ACEI) as well as optimal metabolic control have been suggested to delay or even prevent severe GSD-I kidney disease. The adequacy of metabolic control is usually assessed by glucose, lactic acid, triglycerides, and uric acid blood levels. Obtaining accurate blood lactate levels can be challenging in children whereas plasma alanine level reflecting chronic lactic acidosis could play a role in the assessment of metabolic control.

## Introduction

Glycogen storage disease type I (GSD-I) is one of the most common glycogen storage disorders (1). It is inherited as autosomal recessive condition affecting glucose-6-phosphate metabolism. Two major subtypes are recognized: GSD type Ia, (GSD-Ia) caused by glucose-6- phosphatase (G6Pase) gene mutation leading to G6P deficiency and type Ib (GSSD-Ib) due to SLC37A4 gene mutation (2) due to defective glucose-6-phosphate transporter (G6PT) activity. G6Pase is expressed in liver, kidney and intestine, while G6PT is expressed ubiquitously. In GSD, the final steps of glycogenolysis and gluconeogenesis are blocked leading to an increase in cytoplasmic Glucose-6-phosphate with hepatic and renal glycogen accumulation (3). Several biochemical disturbances are subsequently observed with hypoglycemia upon short fasting and concomitant hyperlactic acidemia, due to the conversion of unutilized energy substrates into lactic acid (3). Recurrent hypoglycemic episodes and hyperlactic acidemia are associated with hyperalaninemia, secondary to the pyruvate aminotransferase reaction and impaired uptake and utilization of alanine by hepatic cells (4). Hypertriglyceridemia and hyperuricemia are also observed in Glucose-6-phosphatase deficiency (5). Diagnosis of GSD-I relies on clinical and biochemical manifestations, in addition to enzymatic and/or genetic testing (5).

Kidney involvement in GSD-I may manifest with proximal and distal tubulopathies. The former is characterized by β2-microglobulinuria, aminoaciduria, phosphaturia (6), hypocitraturia, and hypercalciuria, but a full blown Fanconi's nephropathy is seldom observed (7). Dietary therapy may reverse these abnormalities (8). Restaino et al. (9) described a distal tubulopathy characterized by incomplete distal renal tubular acidosis. Kidney stones have also been described in association with hypercalciuria, hypocitraturia and hyperuricosuria (10).

A more serious kidney complication observed in GSD-I is glomerular hyperfiltration leading to microalbuminuria and subsequently, significant proteinuria, glomerulosclerosis, and kidney failure (11).

Treatment of GSD-I is primarily based on nutritional management to avoid hypoglycemia and to ensure normal neurodevelopment and growth. Liver transplant or combined liver/kidney transplant may be required in severe cases (5).

Angiotensin-converting enzyme inhibitors (ACEI) can minimize kidney hyperfiltration and prevent the development of proteinuria and its sequelae (12). Potassium citrate, allopurinol, hydration, and thiazides have been used, respectively, to manage hypocitraturia, hyperuricemia, and hypercalciuria to avoid nephrolithiasis (5).

Optimal metabolic control, reflected by normal blood glucose level, uric acid, lactic acid, and triglycerides may delay the development of microalbuminuria and GSD-I kidney disease (13). Prevention of kidney injury requires a regular metabolic work-up and a tight control of biochemical risk factors including lactic acidemia. A 24-h glucose-lactate cycle would be a good reflection of metabolic control and dietary compliance, but it is often difficult to obtain in most patients, as it requires hospitalization (12). Furthermore, obtaining an accurate blood lactic acid level may be challenging, especially in infants where false positive elevation of lactic acid level can be secondary to crying or prolonged use of tourniquet. Chronic elevation of lactic acid is associated with increased plasma alanine levels (5), thus making the latter a plausible indicator of poor metabolic control in GSD-I.

We are not aware of any studies assessing hyperalaninemia in GSD 1 patients as marker of metabolic control and early indirect sign of GSD-I kidney disease.

The aim of this study is to evaluate the outcome of kidney disease in a cohort of 32 patients with GSD-I, in addition to their biochemical profile, in particular, alaninemia.

## Materials and Methods

We conducted a retrospective chart review of patients diagnosed with GSD-I followed at the American University of Beirut Medical Center (AUBMC), between 2007 and 2018. The study was approved by the Institutional Review Board at the American University of Beirut (IRB# BIO-2018-0089). The diagnosis of GSD-I was suspected on clinical grounds and confirmed by biochemical, enzymatic and/or mutation analysis. Patients were followed every 3 months at the Inherited Metabolic Diseases Program and the Pediatric Nephrology clinics. Data collected included: age at presentation and diagnosis, clinical manifestations and physical exam findings, growth parameters and body mass index (BMI). The corresponding median Z score for BMI was calculated for each patient according to the Center for Disease Control standards for BMI (normal between 0 and 2 Standard deviations). Blood pressure was measured at each visit, and hypertension was considered for values ≥ 95th percentile.

Besides frequent daily monitoring of blood glucose by dextrostix or by continuous blood glucose monitoring system, regular biochemical assessment, every 3 months, included uric acid levels, blood lactic acid, and triglycerides. Alaninemia, reflecting chronic hypoglycemia episodes, and hyperlactic acidemia, was measured by plasma amino acid chromatography.

Evaluation of kidney function involved measurement of serum, creatinine clearance calculated using the revised Schwartz formula, in addition to measurement of spot urine albumin/creatinine ratio, calcium/creatinine ratio, and citrate/creatinine ratio. In patients who developed proteinuria, microalbumin/creatinine ratio was obtained before administration of ACEI and repeated at 3 and 6 months after initiation of therapy. Effectiveness of treatment with ACEI was defined by the detection of a statistically significant drop in the microalbumin/creatinine ratio. Assessment of optimal metabolic control was defined as: blood glucose > 63 mg/dL, uric acid <7 mg/dL, lactic acid <2.5 mmol/L, triglycerides <530 mg/dL, and BMI between 0 and + 2 standard deviations (SD), based on the definition by the European Study on Glycogen Storage Disease Type 1.

Mean blood levels of uric acid, alanine, lactic acid and triglycerides, and were correlated with the presence of proteinuria. Correlation between BMI and proteinuria was also studied.

Radiological investigations were also recorded for each patient including an ultrasound of the abdomen and the pelvis to check for hepatomegaly, splenomegaly nephromegaly, nephrolithiasis, and gallbladder stones.

### Statistical Analysis

Due to small sample size secondary to disease rarity, normal distribution could not be assumed. Shapiro-Wilk test was used to check for normality. Non-parametric tests were used. The Wilcoxon signed-rank test was applied to compare pre and post ACEI groups. Mann-Whitney U test was used to compare patients with and without albuminuria. A *p* < 0.05 was considered significant. IBM SPSS statistics for Windows, version 25.0 (Armonk, NY, IBM Corp.) was used for statistical analysis.

## Results

GSD Ia was diagnosed in 26 patients and GSD Ib in 6 patients. Age at presentation varied between birth and 7 years with a median of 6 months. The majority (78%) of patients presented before 1 year of age. Consanguinity was present in 75% of cases. Male to female ratio was 3:1. All patients were found to have characteristic doll-like facies, abdominal distension and hepatomegaly. Fifty three percent of the patients presented for hypoglycemic episodes. This was associated with seizures in 22%, while 12% were referred for isolated developmental delay. Splenomegaly was detected in 67% of GSD-Ib patients. One infant (patient 19) presented with epistaxis at the age of 3 months. Half of GSD-Ib patients had a history of recurrent infections. Short stature and failure to thrive were noted at presentation in 31 and 22% of patients, respectively (Table 1). Mean body mass index (BMI) z-score for age upon presentation was 0.16 Kg/m2 and 2.6 kg/m2 at last follow-up. Follow-up duration varied between 1 month and 12 years with a median of 14 months.

**Table 1:** Clinical presentation and kidney outcome of GSD type I patients.

**GSD**		**Clinical features at diagnosis**								**Growth and renal outcome at last follow up**								**Gene test**
	**Patient/gender**	**CG**	**Age at Dg**	**Sz**	**D**	**HG**	**Ht^**a**^**	**Wt^**a**^**	**BMI^**a**^**	**FU duration**	**Ht^**a**^**	**Wt^**a**^**	**BMI^**a**^**	**Hypocitraturia^**b**^**	**Hypercalciuria^**c**^**	**Micro-** **albuminuria**^**d**^	**Outcome**	
Ia	1/M	No	3 m	+	–	+	−2.00	0.2	0.98	12 y	−2	0.02	0.98	+	–	+	GP	
	2/M	Yes	4 m	+	+	+	−2.05	−1.38	−0.23	10 m	−1.2	0.21	1.26	–	–	–	–	
	3/M^a^	Yes	5 y	–	+	–	−1.97	−0.59	1.03	12 y	161*	67.5*	26*	+	+	+	GP, NL	
	4/M	Yes	6 m	–	–	+	−3.10	−1.36	1.21	8 y	−3.6	−1.7	1.01	–	–	–	–	
	5/F	Yes	2 y 2 m	–	–	+	2.50	2.32	1.12	10 m	2.5	2.32	1.12	–	–	–	–	
	6/F	Yes	1 y 3 m	–	+	–	−2.50	−0.66	1.41	7 y 4 m	−2.7	−0.2	1.55	+	–	–	TD	
	7/M	Yes	3 y	–	–	–	−1.40	−1.04	2.07	1 y	−1.15	1	2.7	–	–	–	–	
	8/M	Yes	6 m	–	–	–	−2.20	−1.04	1.12	4 y	−1.9	−0.56	1.48	–	–	+	GP	
	9/M	Yes	4 m	–	–	–	−1.50	−2.2	−1.04	3 y 8 m	−1.7	−0.26	1.48	–	–	+	GP	
	10/M	Yes	1 y 1 m	–	–	+	−2.40	−3.51	−1.23	1 y 2 m	−1.5	−2.2	−1.04	–	–	+	GP	c.247 C > T
	11/F	Yes	2 y 2 m	–	–	–	−3.24	−5.27	−5.18	3 y 10 m	−2.48	−1.53	0.595	–	–	–	–	
	12/M	Yes	3 m	–	–	–	−2.20	−1	0.96	2 m	−3.24	−5.27	−5.18	–	–	–	–	
	13/M	Yes	6 m	–	–	+	0.55	0.9	0.7	1 y 11 m	−2.2	−1	0.96	–	–	+	GP	c.247 C > T
	14/M	No	4 m	–	–	–	−3.80	1	4.18	4 m	−0.7	0.7	1.25	–	–	+	GP	c.247 C > T
	15/M	Yes	3 y	–	–	–	−1.58	−1.89	−1.32	11 m	−3.25	1	3.86	+	–	+	GP	
	16/F	No	1 m	–	–	–	−2.00	1	3.86	6 m	−1.38	−1.26	−0.64	–	–	–	–	
	17/M	Yes	6 m	–	–	+	0.20	1	0.79	2 m	−2	1	3.86	–	–	–	–	
	18/M	Yes	10 m	–	–	+	−2.42	−1.73	−0.54	1 y 10 m	0.2	1	0.79	–	–	–	–	
	19/M	Yes	5 m	–	–	–	0.79	−1.8	−3.13	12 y	−1.3	−0.65	−0.15	+	–	–	LT, ESRD	
	20/M	No	8 m	+	–	+	1.20	−0.43	−2.2	1 y 1 m	−1	−1.6	1.3	–	–	–	–	
	21/F	Yes	5 m	–	–	+	−3.15	−1.92	−0.1	3 y 7 m	1.2	−0.43	−2.2	–	–	–	–	
	22/M	Yes	2 m	–	–	–	−4.18	−2.51	0.05	1 y 7 m	−2.55	−2.26	0.43	+	–	–	TD	
	23/F	Yes	7 m	–	–	+	−2.23	−0.87	0.62	12 m	−3.7	−2.58	−0.21	–	–	–	–	
	24/M	No	4 m	–	–	+	−1.30	−2.32	−2.14	1 m	−2.23	−0.87	0.62	+	+	+	GP, NL	
	25/M	Yes	5 m	–	–	+	−0.87	−1.35	−1.26	1 m	−1.74	−1.34	−0.45	–	–	–	–	
	26/M	Yes	2 m	–	–	–	−2.34	−2.42	−1.49	10 m	−1.4	−0.12	0.8	–	–	–	–	
Ib	27/M	No	1 m	–	+	+	−0.71	0.57	1.29	12 y	175*	101*	32.3*	+	–	+	GP, SMG, RI	p.V236del, c.706-708delGTG
	28/F	Yes	7 m	+	–	+	0.92	0.89	0.51	2 m	−0.87	0.73	1.64	–	–	–	–	
	29/M	No	1 y 9 m	+	–	–	−4.37	−2.63	0.55	11 m	−0.005	0.86	1.23	–	–	–	SMG	
	30/M	Yes	6 m	+	–	+	−2.21	−0.87	0.65	3 y 6 m	−4.37	−2.63	0.55	+	–	–	TD, SMG, RI	c.1348G > A
	31/M	Yes	15 m	–	+	–	−1.47	−0.1	1.01	1 y	−3.23	−0.82	1.81	–	–	+	GP	
	32/F	No	4 m	+	–	–	−1.47	−0.1	1.01	10 y 8 m	−1.6	0.84	2.03	+	–	–	TD, SMG, RI	c.1348G > A

M, male; F, female; Dg, diagnosis; Sz, seizure; D, developmental delay; HG, hypoglycemia; Ht, height; Wt, weight; BMI, body mass index; ACEI, Angiotensin converting enzyme inhibitors; GP, glomerulopathy; TD, tubular dysfunction; LT, liver transplant; NL, nephrolithiasis; ESRD, end stage renal disease; SMG, splenomegaly; RI, recurrent infections;

a height-for-age, Weight-for-age and BMI-for-age Z-scores;

b citrate/creatinine ratio <300 mg/g;

c calcium/creatinine ratio > 0.2 mg;

d microalbumin/creatinine ratio: normal: <30 μg/mg. *height, weight, and BMI for age are listed instead of z-scores for adult patients. Highlighted, patients with elevated microalbumin/creatinine ratio.

### Confirmatory Diagnosis

Confirmatory diagnosis was obtained by liver biopsy, enzymatic assay and/or genetic testing. Molecular testing performed in 20% of cases revealed known mutations: c.247C > T of the G6P gene in 3 GSD-Ia patients, double heterozygous mutation p.V236del, c.706-708delGTG in one GSD-Ib patient while c.1348G > A mutation was identified in 2 others (Table 1).

### Dietary Management

All patients were started on a diet providing daily caloric intake to meet the recommended dietary allowance, with carbohydrates at 60–70% of calories (20). Feeding was provided every 3 h day and night. Nocturnal continuous provision of a glucose polymer via a feeding pump was initiated in 16% of patients up to 1 year of age.

### Kidney Disease Outcome

All patients had nephromegaly documented by ultrasound. Three developed systemic hypertension (patient 3, 19, and 27). Serum creatinine was normal in all patients, except in one patient who had a liver transplant at the age of 10 years (patient 19) and had post-operative complications, reaching end stage Kidney failure needing dialysis at 17 years of age.

Patients with hypocitraturia (31%) and hyperuricemia (28%) were, respectively, treated with potassium citrate and a xanthine oxidase inhibitor, allopurinol. Hypercalciuria was found in 2 patients who developed nephrolithiasis: one at 7 months of age (patient 23) and the other at 15 years of age (patient 3). Both patients had hypocitraturia as well (Table 1) but no hyperuricemia.

Eleven patients (34%) developed microalbuminuria (Table 2) associated with increased GFR in those above 1 year of age. Early age of onset of microalbuminuria, below 7 months, was noted in 4 patients (36%). Treatment with ACEI was initiated upon diagnosis of microalbuminuria leading to a significant reduction then normalization of microalbumin/creatinine ratio at 3- and 6-months follow-up.

**Table 2:** Biochemical profile of GSD type I patients who developed microalbuminuria, and their respective response to ACE inhibitors, at 3- and 6-months interval.

**Patient**	**Age at Diagnosis**	**Uric acid** **mg/dl (P)**	**Alanine** **μmol/l (P)**	**Lactic acid** **mmol/l (P)**	**Triglycerides** **mg/dl** **(P)**	**Proteinuria** **Age**	**GFR**	**ACR** **pre- ACEI**	**ACR** **3 m post- ACEI**	**ACR** **6 m post- ACEI**
1/M	3 m	8.9	594	7.05	980	9 y	339	171	35	21
24/M	4 m	3.4	543	5.42	748	4 m	135	200	37	17
14/M	4 m	5.2	545	6.7	3,174	4 m	97.5	120	63	19
9/M	4 m	6.22	587	6.7	1,260	3 y	217	150	25	16
31/M	15 m	5.8	720	5.2	528	17 m	225.5	89	25	11
8/M	6 m	5.4	623	8	1,250	6 m	76	280	60	24
13/M	6 m	6	742	9	1,367	7 m	85	50	20	12
10/M	10 m	5.8	620	4.6	410	12 m	155	89.2	32	20
15/M	3 y	4	659	2.8	320	3 y 6 m	247	343	36	20
3/M	5 y	4.8	613	1.06	700	5 y	310	252.8	48	18
27/M	1 m	7.6	720	6	210	20 y	175	300	120	29

*P, mean plasma level; y, years; m, months; GFR, Glomerular filtration rate in ml/mn/1.73m^2^(Schwartz equation); ACEI, Angiotensin- Converting Enzyme inhibitor; ACR, Urine microalbumin/creatinine ratio. Reference range: Uric acid: 2.0–5.5 mg/dl; Alanine: reference range: between 1 month and 2 years of age: 143–439 μmol/l; between 2 and 20 years of age: 152–547 μmol/l; Lactic Acid: 0.55–2.20 mmol/l, triglycerides: desirable <150 mg/dl. ACR: normal: <30 μg/mg creatinine*.

In patients who developed microalbuminuria (Table 2), suboptimal metabolic control associated with poor dietary compliance was reflected by hyperlactic acidemia (91%), hypertriglyceridemia (82%), and hyperuricemia (27%), while hyperalaninemia was observed in all these patients (100%).

Biochemical parameters including mean blood uric acid, alanine, lactic acid and triglycerides levels were elevated in all GSD-1 patients who developed proteinuria were compared to those who did not (*p* < 0.05). In contrast, no significant correlation was found between BMI and appearance of microalbuminuria (*p* = 0.58 at presentation and *p* = 0.08 at follow-up). The demographic and biochemical profile of GSD patients who did not develop proteinuria are reported separately in Table 3.

**Table 3:** Metabolic phenotype of GSD type I patients unaffected by microalbuminuria.

	**Patient/gender**	**Age at diagnosis**	Uric acid^*^	Alanine^*^	Lactic acid^*^	Triglycerides^*^	**Follow up duration^**a**^**
**GSD type Ia**	16/F	1 m	4.3	683	3.2	195	6 m
	22/M	2 m	3.1	357	1.7	175	1 y 7 m
	26/M	2 m	3.7	428	1.3	124	10 m
	12/M	3 m	2.8	251	1.19	338	2 m
	2/M	4 m	4	452	2	273	10 m
	19/M	5 m	5	500	1.05	129	12 y
	21/F	5 m	3.8	168	3.1	368	3 y 7 m
	25/M	5 m	4.6	173	2.5	857	1 m
	17/M	6 m	4.2	215	1.9	83	2 m
	4/M	6 m	3	420	2.8	491	8 y
	23/F	7 m	3.5	447	2.3	420	1 m
	20/M	8 m	5.1	480	2.6	926	1 y 1 m
	18/M	10 m	4.8	327	1.6	735	1 y 10 m
	6/F	1 y 3 m	5.7	410	2	307	7 y 4 m
	5/F	2 y 2 m	8.3	452	2.6	147	10 m
	11/F	2 y 2 m	3.8	168	3.01	368	3 y 10 m
	7/M	3 y	4.1	210	0.94	289	1 y
**GSD type Ib**	28/F	7 m	4.8	237	2.48	416	2 m
	29/M	1 y 9 m	8.8	231	3.54	241	1 m
	30/M	6 m	2.9	185	1.3	147	3 y 6 m
	32/F	4 m	4.7	530	2.6	310	10 y 8 m

* mean plasma levels.

a *since diagnosis. Reference range: Uric acid: reference range: 2.0–5.5 mg/dl; Alanine: reference range: between 1 month and 2 years of age: 143–439 μmol/l; between 2 and 20 years of age: 152–547 μmol/l; Lactic Acid: 0.55–2.20 mmol/l, Triglycerides: desirable <150 mg/dl, Microalbumin/creatinine ratio: Increased: > 30 mg/24 h*.

## Discussion

Chronic kidney disease is a common complication observed in GSD-I, at times leading to kidney failure following a silent phase (7). The incidence of kidney involvement is variable as described in some reports.

In a study by Visser et al. (14), 70% of young adult patients experienced hyperfiltration and microalbuminuria. Glomerular hyperfiltration and increased renal plasma flow are found in the early stages of kidney dysfunction in GSD-I patients (12). The cause of the kidney injury is still unknown, but several mechanisms have been described. Activation of various pathways, in particular, glycolysis, *de novo* lipogenesis, and activation of the renin angiotensin system may lead to the progression of kidney disease (7). Thus, kidney damage may develop after a period of “silent” glomerular hyperfiltration, with microalbuminuria, proteinuria and systemic arterial hypertension. These will subsequently lead to renal failure in a considerable number of patients (15). Although kidney disease seems inevitable in GSD 1 patients, the role of metabolic control is controversial, since Glucose 6 phosphate is absent in the glomeruli and strict metabolic control can affect usually the occurrence of tubulopathy more than glomerulopathy. Therefore, even with optimal metabolic control and ACEI therapy, some patients would still progress to kidney failure (12). Recently, Okechuku et al. (16) demonstrated that tight metabolic control in addition to ACEI therapy improves GSD1 kidney disease and may delay its occurrence.

We report our 12-year experience in 32 patients with GSD types I-a and I-b and describe the main clinical presentation and their renal outcome. Nephromegaly was observed in all patients, reflecting glycogen accumulation in cortical tubules (8). Kidney injury was found in one third of the patients who developed microalbuminuria (34%), requiring early treatment with ACEI. Glomerular hyperfiltration, identified in 63% of microalbuminuric patients, occurred in those who developed microalbuminuria after 1 year of age similar to other reports in the literature (9). In contrast to a study by Martens et al. (17), early appearance of microalbuminuria in our patients may have been due to the late diagnosis of GSD and/or poor metabolic control, as reflected by high alanine, lactic acid, and triglyceride levels. Chen et al. (18), reported kidney failure in 40 percent of their cohort and one death due to kidney failure while in our series, only one patient progressed to chronic kidney disease requiring dialysis at the age of 18 years. Patients who are well-controlled are more likely to have preserved renal function and late, if any, appearance of microalbuminuria. Control of uric acid is very important among the metabolic markers. Indeed, uric acid exerts a deleterious effect on tubular cells by inducing epithelial-to-mesenchymal transition (EMT) in the proximal tubules. Hyperuricemia in GSD-I can be explained by a dual mechanism of inhibition of uric acid tubular excretion by lactic acid along with an increased degradation of adenine nucleotides (19). The treatment of hyperuricemia with allopurinol has been reported to avoid kidney injury (20). Persistent hyperuricemia was observed particularly in 2 patients with good metabolic control, despite treatment with a xanthine oxidase inhibitor (5).

As reported by Weinstein et al. (10), optimal metabolic control did not seem to prevent hypocitraturia and tubular dysfunction: half of the patients developed hypocitraturia after a variable time of evolution, ranging between 21 months and 12 years. This might be possibly explained by the development of an incomplete distal tubular acidosis (9). Although the exact mechanism for kidney stone formation in GSD-1 is not clear, chronic acidosis, hypocitraturia, hypercalciuria and hyperuricemia are thought to be contributing factors. Reistano et al. (9), reported that renal calculi were associated with hypercalciuria in most patients with GSD-1 (9). In our study, only two patients developed kidney stones with hypercalciuria, with no chronic hyperuricemia as it was controlled by Allopurinol. Hypertension was found in 3 out of 11 patients with microalbuminuria, which might suggest that hypertension did not play a role in the glomerular injury observed in the remaining cases. One third of the patients had short stature which can be linked to poor metabolic control (21). The relation between obesity and microalbuminuria has been demonstrated in hypertensive and/or diabetic patients (22). Nevertheless, controversies exist whether a high BMI by itself is predictive for microalbuminuria occurrence (18). In this study, we found no correlation between BMI and microalbuminuria in GSD-I patients.

Genetic study performed in 20% of the patients revealed known disease-causing mutations for both GSD Ia (23) and Ib patients (24, 25). To date, genotype-phenotype correlation studies do not show a strict correlation in GSD- Ia (26) nor in GSD-Ib (27, 28). In our series, c.247C > T mutation of the G6P gene was detected in three GSD-Ia patients with glomerulopathy while 2 GSD-Ib with tubular dysfunction had c.1348G > A mutation and did not develop glomerulopathy after a follow-up period between 3 years 6 months and 10 years 8 months. However, we cannot draw any strong conclusion regarding phenotypic kidney outcome in correlation with a specific genotype in this series.

It is known that the development of kidney disease in GSD- I patients might be delayed by good metabolic control in addition to medical therapy with ACEI (16). In our study, in addition to blood uric acid, lactic acid, and triglyceride levels, we used plasma alanine as a marker of the metabolic status. Chronic elevation of mean plasma alanine level was detected in all patients with poor metabolic control who developed microalbuminuria. To our knowledge, this is the only study where alanine was assessed to determine the metabolic status, in association with other known biochemical markers. We believe that the role of plasma alanine as an early marker to identify metabolic disturbances should be determined in future studies in GSD-I patients. Early elevation of plasma alanine might help to identify patients at risk and hence, to adapt their diet and/or treatment.

## Conclusion

Plasma alanine, reflecting lactic acidemia, seems to be a promising marker, reflecting metabolic control, that may help in the follow-up of GSD-I patients. Further larger studies are still needed to identify the role of each biomarker in the progression of GSD-I kidney disease in order to optimize patient's outcome and achieve therapeutic goals.

## Data Availability Statement

All datasets generated for this study are included in the article/supplementary material.

## Ethics Statement

The studies involving human participants were reviewed and approved by Institutional Review Board at the American University of Beirut. Written informed consent from the participants' legal guardian/next of kin was not required to participate in this study in accordance with the national legislation and the institutional requirements.

## Author Contributions

PK and BA contributed to the conception and design of the study and data analysis. ABas, PK, and BA contributed to data collection. All authors wrote sections of the manuscript and contributed to manuscript revision, read, and approved the submitted version.

## Conflict of Interest

The authors declare that the research was conducted in the absence of any commercial or financial relationships that could be construed as a potential conflict of interest.
